# Intestinal helminthic parasites of rodents in the central region of Iran: first report of a capillariid nematode from *Dryomys nitedula*

**DOI:** 10.1186/s13104-020-05304-x

**Published:** 2020-09-29

**Authors:** Sina Mohtasebi, Aref Teimouri, Iraj Mobedi, Alireza Mohtasebi, Hamed Abbasian, Mohammad Javad Abbaszadeh Afshar

**Affiliations:** 1 Department of Medical Parasitology and Mycology, School of Public Health, Tehran University of Medical Sciences, Tehran, Iran; 2 Department of Parasitology and Mycology, School of Medicine, Shiraz University of Medical Sciences, Shiraz, Iran; 3 Department of Environmental Health, Shahid Beheshti University of Medical Sciences, Tehran, Iran; 4 Department of Medical Parasitology and Mycology, School of Medicine, Jiroft University of Medical Sciences, Jiroft, Iran

**Keywords:** Rodent, Helminth parasites, *Capillariidae*, *Dryomys nitedula*, Iran

## Abstract

**Objectives:**

Rodents play an important role to spread zoonotic diseases through society. The current study was carried out to collect informative data on the intestinal helminthic infections of wild rodents in Taleqan County, Alborz Province, the center of Iran, emphasizing their zoonotic aspects.

**Results:**

Sixty-two killed rodents by local farmers belonging to five species were collected, among which 24 were identified as *Mus musculus*, 15 as *Meriones persicus*, 12 as *Meriones libycus*, 10 as *Apodemus witherbyi*, and 1 as *Dryomys nitedula*. Of them, 30 (48.4%) were infected with at least one helminth species. Rodents were infected with *Hymenolepis diminuta* (42%), *Syphacia obvelata* (21%), *Hymenolepis nana* (17.7%), *Heligmosomoides polygyrus* (9.6%), *Trichuris muris* (8%), and as well as a capillariid nematode that was isolated for the first time from *D. nitedula* in Iran. The findings of the present study revealed a significant intestinal helminthic infection of rodents in Taleqan County. Improving hygiene practices, and making a preventive attitude can be helpful to reduce the hazards of rodent-borne diseases in the area where humans, livestock, and synanthropic rodents are living close to each other.

## Introduction

Rodents, as the most diversified order of mammals, are considered as reservoirs of several zoonoses. These small mammals play an important role in the transmission and spreading of zoonotic diseases, through harboring many pathogen agents, including helminthic species [1].

Due to the different ecological, climate, and zoogeographical conditions, Iran is considered a hotspot for rodents [1, 2]. There are various reports of rodent parasite infectivity in various parts of Iran, while most of them have been reported zoonotic species, such as *Hymenolepis nana*, *Hymenolepis diminuta*, and *Syphacia obvelata* [3–15]. However, the rodent’s parasite fauna in each ecological setting may differ due to environmental variations around the country and thus further studies in areas with different ecological settings may seem required.

Due to the impact of rodent-borne diseases on human and livestock health status, the study of rodent’s parasites in every geographical area could be a fundamental step to set up an effective control program and pave the way to improve the general health status. Despite previous studies on rodent’s endoparasites in Iran, especially helminth species, there is still less knowledge about helminths infection, distribution, and diversity in most regions of Iran [14]. On the other hand, since rodents are considered as agricultural pests that reduce the yield of agricultural farms and spoil stored food [16], local farmers control their population by eliminating them. Our study was carried out on the killed rodents by local farmers of Alborz Province. This study aimed to identify the intestinal helminths fauna of these small mammals and their zoonotic implications.

## Main text

### Methods

#### Study area

Taleqan County, located in the mountains of Alborz Province, the center of Iran (36º 83′ 93" N and 54º 44′ 44" E), is known for its mild, sunny summers and extremely cold winters. Horticulture, agriculture, and animal husbandry are among the main economic activities in Taleqan, with a large proportion of the population engaged in these sectors.

#### Rodent collection and identification

Sixty-two killed rodents by local farmers in horticultural, agricultural, and animal farms of Taleqan region were collected from 2018 to 2019. Collected rodents were transferred to the Animal Unit of School of Public Health, Tehran University of Medical Sciences. The identification of the species was completed using a valid identification key [17]. All the procedures of the current study were approved by the research ethics committee of Tehran University of Medical Science, Tehran, Iran.

#### Isolation of parasitic helminths from rodent intestines and identification

The parasites were isolated from rodents’ intestines. At necropsy, their alimentary canals were removed and the contents of each part were washed using phosphate-buffered saline (PBS). Then, the lining membrane of intestines was gently scraped with a scalpel blade and the contents were examined carefully for the presence of helminths in the large Petri dishes containing PBS, using a stereo microscope. The helminths were collected from the scraped materials and preserved in buffered 10% formalin and 5% glycerin alcohol. Isolated helminths were cleared with lactophenol and stained by Formaldehyde-Alcohol-Azocarmine-Lactophenol (FAAL) and carmine staining solutions and then were mounted, using Canada balsam. The identification of the isolated helminths was completed based on appropriate systematic keys [18–20]. Morphological characteristics of the recovered helminth were drawn carefully, if necessary, using a camera lucida equipped microscope, at 400 × magnification. For each animal, the feces samples were prepared for the direct microscope examination to diagnose the parasite's ova.

### Results

During the study period, sixty-two rodents belonging to five species were collected, among which 24 (38.7%) were identified as *Mus musculus*, 15 (24.2%) as *Meriones persicus*, 12 (19.4%) as *Meriones libycus*, 10 (16.1%) as *Apodemus witherbyi* and 1 (1.6%) as *Dryomys nitedula*. The microscopic examination of gastrointestinal tract contents revealed the presence of six species of different helminths. At least one species of the intestinal helminths was found in 30 of the examined rodents resulting in a prevalence of 48.4%. The most percentage of infection was found in *M. musculus* (54.1%) (Table 1). Also, intestinal helminthic infections of collected rodents, based on rodent species, are embedded in Table 1. *H. diminuta* (42%) was the most common species in all collected rodents. *S. obvelata* (21%) (6 males and 49 females) and *H. nana* (17.7%) were the other common species, respectively. Moreover, 9.6 and 8% of the infectivity rate was recorded for *Heligmosomoides polygyrus* (31 males and 25 females) and *Trichuris muris* (23 male and 47 females)*,* respectively*.*

**Table 1:** Abundance of isolated intestinal helminths according to rodent species (n = 62)

Rodent species	No. of collected rodents	No. of infected rodents (%)	Isolated helminths					
			*Syphacia obvelata*	*Heligmosomoides polygyrus*	*Hymenolepis nana*	*Hymenolepis diminuta*	*Trichuris muris*	Capillariid nematode^a^
*Mus musculus*	24	13 (54.1)	5	2	4	10	2	–
*Meriones persicus*	15	7 (46.6)	3	1	2	6	–	–
*Meriones libycus*	12	6 (50)	4	2	4	7	2	–
*Apodemus witherbyi*	10	3 (30)	1	1	1	3	1	–
*Dryomys nitedula*	1	1 (100)	–	–	–	–	–	1
Total^b^	62	30 (48.4)	13 (21%)	6 (9.6%)	11 (17.7%)	26 (42%)	5 (8%)	1 (1.6%)

^a^First report of a capillariid nematode from *Dryomys nitedula* in Iran

^b^There were also some cases of coinfection with multiple helminthic species

**Fig. 1:**
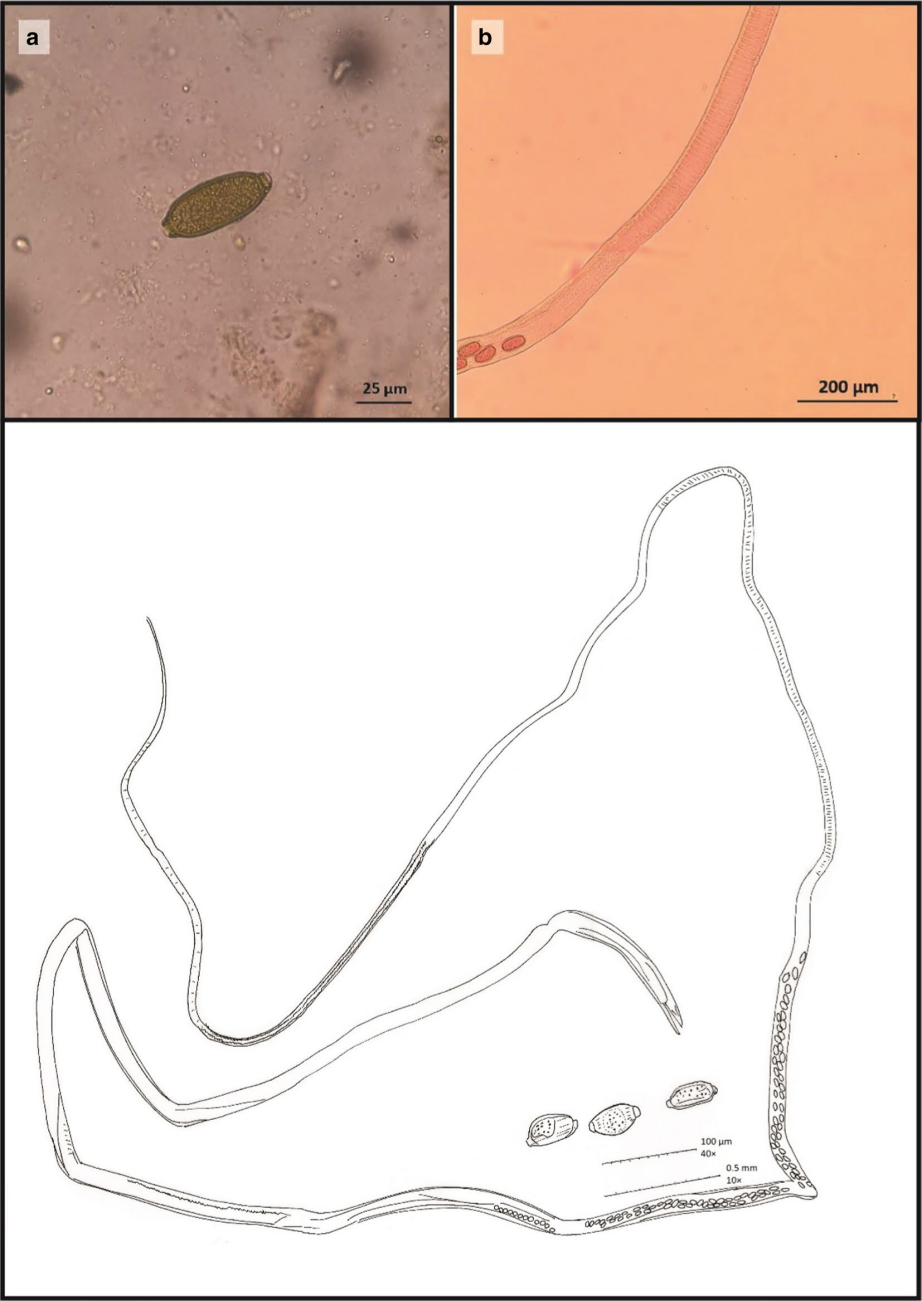
The capillariid nematode isolated from *Dryomys nitedula* for the first time in Iran. **a** Egg (400 ×), **b** Female worm, Stichocytes are present (100 ×), **c **Camera lucida drawing

**Fig. 2:**
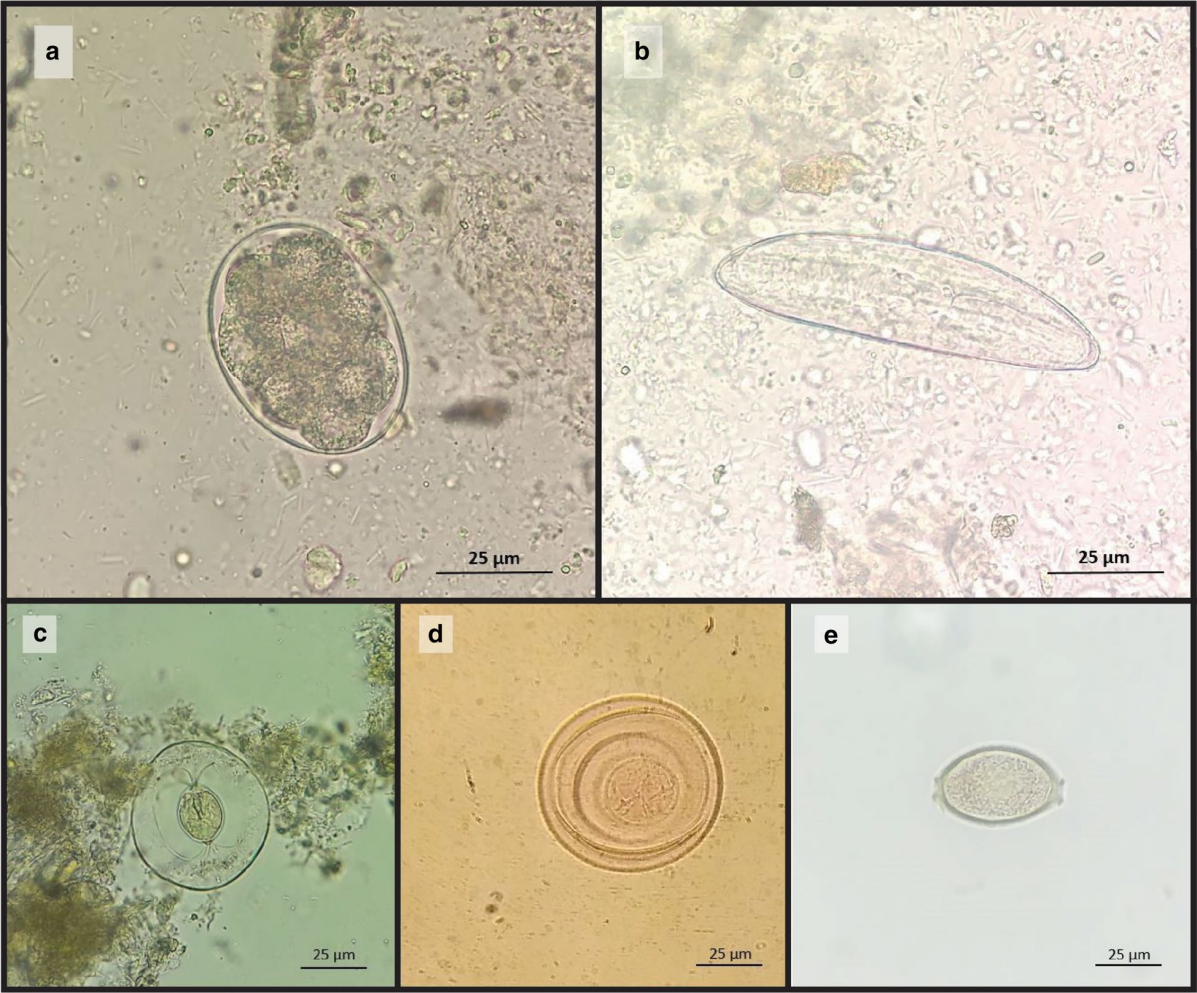
Helminths ova from intestinal contents of collected rodents from Taleqan (400 ×). **a** *Heligmosomoides polygyrus*, **b** *Syphacia obvelata*, **c** *Hymenolepis nana*, **d** *Hymenolepis diminuta*, **e** *Trichuris muris*

In this study, a female capillariid nematode was detected in *D. nitedula* for the first time in Iran. The microscopic and camera lucida images related to this capillariid nematode have been shown in Fig. 1. The recovered nematode was about 16 mm in length and 0.1 mm in width with a clear stichosome. The eggs were measured approximately 57 × 26 µm, and as drawn in Fig. 1c, the eggs shell had an irregular pattern. Furthermore, microscopic images of the other isolated helminths ova are shown in Fig. 2.

### Discussion

Determining the rodent parasites fauna in various zoogeographical zones can complete the puzzle of information, concerning the possible potentials for transmission of zoonotic helminths to humans and livestock in the given areas [21]. In the present study, 48.4% of the rodents were infected with at least one helminth species. This finding is consistent with most studies carried out in various regions of Iran, which report a high infectivity rate of helminthic parasites in different species of rodents [4, 8, 11, 22–26]. In a comprehensive study carried out by Moradpour et al. [14] on rodent’s helminth parasites in three climate zone of Iran, thirteen species of rodents were recognized, of which the *M. musculus *was the most prevalent which is concordant to our finding. They report 43% of the infectivity rate among the 253 investigated rodents. Regarding the role of rodents in the spread of zoonotic parasitic agents in the environment and their risk for public health and the high infectivity rates of zoonotic helminthic species in rodents reported in Iran, it seems a comprehensive control plan and a preventive approach are required.

As far as the authors know, this study is reporting a capillariid nematode from *D. nitedula* (Forest Dormouse) for the first time in Iran. Previously, helminths fauna of *D. nitedula* has been studied in Belarus and genus *Armocapillaria*, as a subgenus of *Aonchotheca* (Trichinelloidea: Capillariidae), was reported [27] while in Iran few studies have been carried out on *D. nitedula* parasites. Tapeworm infection was reported from *D. nitedula* in Kerman Province [11] while no parasitic infections were reported from dormice in Khorasan Province [23].

Based on our knowledge the present study is the first survey on rodent parasites in Alborz Province. In this study, 6 helminth species were detected from alimentary canals of 5 rodent species. Among these 6 species, *H. nana* and *H. diminuta* and *S. obvelata* have zoonotic importance [4]. In the present study, *H. diminuta* was the most prevalent helminth detected in different rodent species, similar to other conducted studies in Kohgiluyeh and Boyer-Ahmad and Ardabil Provinces [4, 26]. Human infection with, *H. diminuta* or the "rat tapeworm" occurs after the accidental ingestion of some insects such as immature fleas, flour moths or beetles, and cockroaches that harbor cysticercoid stage of the parasite in their body cavities. The *H. diminuta* human infection cases have been reported sporadically from Iran [28–31].

Several studies have confirmed the zoonotic potential of *S. obvelata*. The first report on human infection with *S. obvelata* recorded in 1919, when the eggs and 2 mature females of the parasite identified in a child from Philippines [32]. Since then, other cases of human infection by *S. obvelata* have been reported rarely around the world [33, 34]. Furthermore, there are records *S. obvelata* eggs being found in mummified human bodies from Nubia dated back to 300–700 years Before Christ [35]. Referring to the carried-out studies on rodents in different parts of Iran, while this species was one of the most common [4, 14, 23], no human case has been recorded in Iran to date. Another prevalent species in this study was *H. nana* (dwarf tapeworm) which is potentially transmissible to humans. Although the prevalence of *H. nana* in humans has fallen since 1970, it remains relatively common in the rural areas of Iran [36]. Given the high prevalence of these species in the investigated rodent, it seems an investigation on human intestinal parasites, and also, a comprehensive control program is required in Taleqan County.

In the current study, five rodent species were examined for intestinal helminthic parasites. Among them, the most infectivity rate was found in *M. musculus* which is consistent with the results of Moradpour et al. [14]. Helminth infections of house mice have been studied in different parts of Iran and *H. diminuta* and *S. obvelata* have been reported as the most common species [37, 38]. However, in some studies around the world *H. nana* was reported as a common species in *M. musculus* [39, 40]. The high infectivity rate of *M. musculus,* and on the other hand, its synanthropic behavior has made this species more potentate to transmitting rodent-borne zoonotic diseases to humans. Taleqan County is a common passageway of wolves, jackals, foxes, otters, and stray dogs [41]. Rodents could play the role of intermediate and paratenic hosts for parasites that infect these carnivores, including the important zoonotic agents of Toxoplasmosis, Hydatidosis, and Toxocariasis [42–44]. Therefore, monitoring of such parasites in rodents and control of them could reduce the risks for human and veterinary public health.

### Conclusion

Our findings highlighted the significant intestinal helminth infections of rodents in Taleqan County. The results revealed that the rodents of Taleqan County are infected with various helminth infections that some of which can be transmitted to humans and may pose a risk to human health. For better management of rodent-borne diseases, it is necessary to seriously consider the role of rodents in spreading infectious diseases in this area. Improving hygiene practices, and making a preventive attitude can be helpful to reduce the hazards of rodent-borne diseases in the area where humans, livestock, and synanthropic rodents are living close to each other. It is necessary to conduct more comprehensive and multidisciplinary studies to better understanding the occurrence of rodent-borne diseases in this area and also throughout Iran.

## Limitations

This work was carried out as a preliminary study at a single site. In this study, for respect to environment and wildlife, we just investigated the killed rodents by local farmers, therefore the reported rodent’s species may not depict the rodent’s fauna in the study area. The identification of capillariid nematodes is based on the male characteristics, although we recovered only a female capillariid nematodes from *D. nitedula*. Therefor our identification was only at the family level.

## Data Availability

All data generated or analyzed during this study are included in this published article. The original datasets are available upon request to the corresponding author.

## Abbreviations

FAAL: Formaldehyde-alcohol-azocarmine-lactophenol
PBS: Phosphate-buffered saline
